# The mental health detention process: a scoping review to inform GP training

**DOI:** 10.3399/BJGPO.2022.0061

**Published:** 2022-10-19

**Authors:** Paula Houton, Helen Reid, Gavin Davidson, Gerard Gormley

**Affiliations:** 1 Centre for Medical Education, Queen’s University Belfast, Belfast, UK; 2 School of Social Sciences, Education and Social Work, Queen's University Belfast, Belfast, UK

**Keywords:** general practice, general practitioners, mental health detention, mental health, training

## Abstract

**Background:**

GPs are often faced with deciding whether or not a patient may require detention for assessment in hospital under mental health legislation. This can be a complex and daunting process. Despite this, GPs and most other professionals receive limited formal training.

**Aim:**

To map and review the current literature on training in mental health detention processes. These insights are vital to inform the further development of meaningful educational approaches.

**Design & setting:**

A systematic scoping literature review was conducted to identify what is known about how best to develop training in this area.

**Method:**

Arksey and O’Malley’s framework was used to select, chart, and analyse articles from across six electronic databases. A total of 1136 articles were included in the initial screening phase and 183 articles were included in the full-text screening phase. Key themes were derived using an iterative and thematic approach. A personal and public involvement (PPI) group was set up for this project and other stakeholders in the mental health detention process were consulted about the findings.

**Results:**

Fifty-two articles were included in the final review. Professionals consistently highlighted unmet training needs and difficulties with the process. There were identified needs for practical, interdisciplinary training, including discussion of complex cases, and opportunities to learn from those with direct experience.

**Conclusion:**

This work is foundational for the development of meaningful educational approaches around mental health detention processes. A strong research base will inform and strengthen training with the ultimate aim of improving patient care.

## How this fits in

There is a recognised clinical need for GPs to have training opportunities to support preparing for complex mental health crisis assessments in the community. Findings from the review confirm a gap in training and also highlight how research in this area is lagging behind clinical need. This review serves as a foundation for the development of future meaningful training approaches for professionals involved in the mental health detention process.

## Introduction

Mental health detention processes are complex and emotive for all involved.^1–3^ They involve balancing respect for patient autonomy and best interests against deprivation of liberty and human rights. Despite these challenges, there are times when a mental health detention is necessary to ensure best patient care. GPs encounter these situations infrequently, yet are expected to be confident and competent using mental health legislation when necessary. Alongside practical issues surrounding necessary paperwork, the clinical decision-making process can be complex and challenging.^4,5^ There is the additional complexity of coordinating a prompt, interdisciplinary team response, while maintaining the provision of safe clinical practice.^2,6^ This high-stakes assessment can have a profound, lasting impact on the patient, carers, and all involved.^1,7–9^

Training is essential to ensure GPs are prepared to deal with this mental health crisis in the community. It is therefore concerning that many GPs report gaps in training.^10^ This apparent disparity between expected GP competencies and training was the initial driver behind this research. It would seem, however, that training in this area is suboptimal across professional groups.^2^ Experiential learning approaches have been successfully implemented for other medical emergencies but are underutilised in mental health crises. Despite a recognised clinical need for training improvements, to date education in this area has received limited attention.

This study sought to explore the evidence base to identify factors relevant to developing a meaningful, educational approach in this area. Identifying how best to address training gaps offers potential to better prepare professionals for this crisis situation and ultimately improve patient care. The aim of this scoping review was to identify what is known in the literature about developing training for professionals involved in the mental health detention process.

## Method

### Research team

The research team included an academic GP trainee, two academic GPs, an academic social worker, a health specialist librarian, and three PPI advisers.

### Methodology framework

This scoping literature review was conducted using Arksey and O’Malley’s six-stage framework.^11,12^ Steps in the Preferred Reporting Items for Systematic Reviews and Meta-Analyses extension for scoping reviews (PRISMA-ScR) guidance were completed.^13^

#### Identifying the research question

The ‘population, situation tool’^14^ was used to develop the research focus, with preliminary searches iterating the final review question. Initial focus on GPs was extended to include interdisciplinary colleagues. Limiting searches to primary care settings was excluding potentially relevant articles, therefore the search was expanded to include literature spanning primary and/or secondary care contexts. The final scoping review question was: what is known about how best to develop training for professionals involved in the mental health detention process?

#### Identifying relevant studies

There were limited articles if the search was refined to education or training, or to primary care settings. Therefore an initial broad search strategy was maintained to capture articles addressing mental health detention processes across professional groups and settings. Table 1 gives an overview of the approach. The authors discussed and selected the electronic databases and search terminology (Table 2). In December 2019, six electronic databases were searched: PsycINFO (1806–2019), Social Policy & Practice, CINAHL, Web of Science, Medline (1946–2019), and Embase (1974–2019). Web of Science was restricted to article titles and Social Policy & Practice was restricted to journal articles. No other limitations were applied. Searches identified 1136 articles, which were imported into Covidence systematic review software for screening.

**Table 1.** Outline of approach for database searches

Steps	Description
1. Mental health detention process	Articles that included terms related to the mental health detention process were searched first
2a. Education or training terms	Then articles were searched that included terms related to education, training, or specific training approaches, which were found while doing background reading
2b. Stakeholder terms	Searches were also undertaken for articles linked to each of the respective stakeholders involved in the mental health detention process
3. Combined search	Then searches were undertaken that combined steps 1 AND (2a OR 2b)

**Table 2.** Search terms used for PsycINFO. These terms were adapted for use across all databases.

Category	Terms
Mental health detention	commitment (psychiatric) or involuntary treatment or mental health detention or compulsory detention or compulsorily detained or mental health* or detention or compulsory power or involuntar*commit* or civil commitment or mental health emerg* or mental health cris* or mental health act or mental health legislation or mental health law or mental health order
Education	train* or educat* or teaching or medical education or nursing education or social work education or (paramedic* or ambulance*)adj3(educat* or train*) or (psychiatrist*adj3 (train* or educat*) or psychiatric training or (police* adj3 (train*or educat*) or clinical education or mental health act training or mental capacity act training or simulation or patient simulation* or simulation training or mental health simulation* or simulation work or respond train* or Maudsley or forum theat* or (**recognising and assessing medical problems in psychiatric settings* or RAMPPS)
Stakeholder	general practitioners or family practitioner* or family doctor* or medic* or doctor or approved social work* or approved mental health professional or social work* or (*community mental health care team** or CMHT) or community health worker or (psychiatric) community worker* team* or nursing or emergency medical technician* or (paramedic* or ambulance*) or psych* or psychiatrist* or police* or patient

#### Study selection

Two authors independently screened 895 titles and abstracts (after removing 241 duplicates). Where there was any uncertainty around inclusion, a third reviewer was involved. This iterative process with frequent team research meetings enabled consensus around inclusion and exclusion criteria for the full-text phase (Table 3).

**Table 3.** Inclusion and exclusion criteria at the full-text phase

Inclusion	Exclusion
*Education/training AND Mental Health (MH) detention process OR MH detention legislationOR*Unmet training needs AND MH detention process OR MH detention legislationOR*Problems/difficulties with the application for MH detention process that could potentially be addressed through training	*Not relevant to the MH detention process OR MH detention legislation*Not relevant to education/training approaches or unmet training needs*Study is clearly unrelated to scoping review question

**Figure 1.**
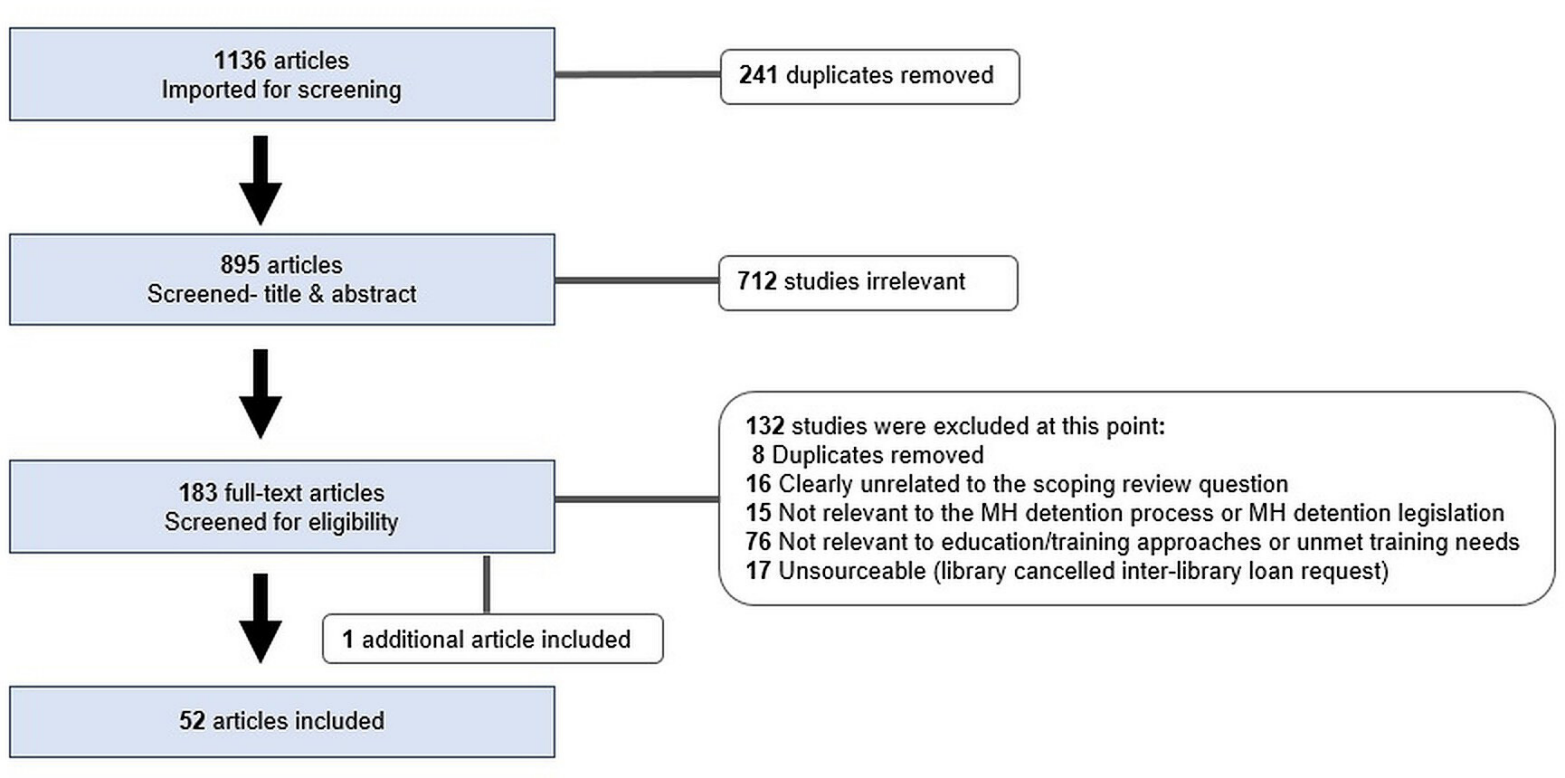
PRISMA diagram: overview at the end of the full-text screening phase. MH = mental health.

Full-text articles (*n* = 183) were considered twice, initially for overall content, subsequently for determining inclusion eligibility. Any articles with inclusion uncertainty were resolved through wider research team discussion, where agreement was also made to include an additional article (meeting inclusion criteria) referenced in an included article. Fifty-two articles met criteria to proceed to data extraction.^1,2,6–10,15–59^ This process and specific reasons for exclusion are presented in Figure 1.

#### Data charting

Data extraction tables were used to summarise key article information including title, source, geographical context, year of publication, and key themes and quotes relevant to the review question. At this point articles were divided into one of four emergent categories: formal mandatory educational approaches;^15–20^ less formal educational approaches;^21–32,50^ mental health legislation;^10,33,34,36–54,49,52,54^ and unmet training needs and wider difficulties with the process.^1,2,6–41–48,51–48,51,53,55–59^

#### Collating, summarising, and reporting results

The included articles were quantitatively and qualitatively analysed. Geographical spread, articles by type, and professional group were considered. The key messages relating to the scoping review question were considered and summaried. An iterative approach was used to develop themes representing the evidence from in-scope articles. In each section current practice was considered and suggested factors for training development were noted.

#### Consultation exercise

This optional methodological step was conducted to identify how literature findings sat with professionals involved. GPs (*n* = 4), approved social workers (*n* = 2), psychiatrists (*n* = 3), paramedics (*n* = 2), and police officers (*n* = 5) were consulted, who each participated in one of four online, interdisciplinary consultations.

#### Personal and public involvement

The authors met PPI advisers on five occasions throughout the research, with further email correspondence. They considered the research approach and were invited to read and comment on included articles. They also observed and subsequently discussed stakeholder consultations.

## Results

### Overview of articles

Included articles were published between 1983 and 2017. They included 31 empirical articles^2,7–18,20,22–26,28–37,40,42–44,46–56,59,42–44,46,48,51,55,56,59^ and 21 descriptive, review, or commentary articles.^1,6,16,17,19,21,27,30,32,38,39,41,45,47,49,50,52–57,58,58^ Geographical spread included the Republic of Ireland,^9,28,47,48,59^ UK,^1,2,6–10,15–20–38–44–49,50–57,38–44,46,49,50,52,54–57^ Canada,^27,32^ US,^21,25,29–37,45,51,58,58^ Australia,^26^ and South Africa.^53^ The range of professional groups involved included GPs,^9,10,28,36,42,59^ medical students,^27^ psychiatrists,^23,29,34,37,38,49–58,58^ emergency medicine doctors,^21^ unspecified ‘doctors’,^22,33,39,45,52,54^ nursing students,^26^ mental health nurses,^15,16,56^ approved social workers,^1,20,35,40,41,43,57^ approved mental health professionals,^6–17–19–19,46^ paramedics,^2^ and police.^25,30–55,55^

### Formal mandatory educational approaches

These articles were UK-based, where training for ASWs and AMHPs is mandatory before undertaking these specialist roles. Five articles focused on educational needs and formal training of professionals making the transition to becoming AMHPs under new legislation in England and Wales.^15–19^ The remaining article considered assessment approaches in formal ASW training courses.^20^ These articles highlighted widespread variation in mandatory education and training, even within jurisdictions sharing common mental health legislation. One article mentioned a 6-month, intensive course designed to achieve the minimum 600 hours of learning through a combination of clinical placement, direct teaching, private study, and supervision.^19^ In contrast, another article described a training pathway integrated into a university master’s programme.^18^

These articles offered many suggestions for training development. They stated the importance of maintaining a patient-centred approach and recognising the emotional impact of this acute work on individual professionals.^16,19^ Other factors included potential benefits of interdisciplinary training and the importance of regular training updates.^16,17^ It was also suggested that training and assessment processes should ideally prepare professionals for practical, real-life knowledge application.^20^

### Less formal educational approaches

Included literature illustrated gaps in formal, mandatory training for GPs and professional colleagues. A number of ad-hoc training approaches were identified, which were implemented in response to practical need. Several articles mentioned development of teaching sessions on mental health legislation in response to clinical need.^21–23^ One described the development of a learning resource to prepare frontline staff for changes in mental health legislation.^24^ The literature also described a training programme delivered to police officers incorporating patient perspectives, crisis intervention, and mental health law.^25^ Overall, these findings suggested that given the lack of more structured training, even short 20-minute online courses could help improve knowledge.^21^

It was suggested that training should begin in undergraduate curricula with scope for interdisciplinary teaching.^26,27^ Educational resources were shown to be useful if they were practical and flexible to suit different learning styles and availability; however, the content needed to be at the correct level and at times role-specific.^24^ Of note, a questionnaire among Irish GPs suggested that informal training through discussions with colleagues may be as effective as formal educational approaches.^28^ Another study suggested that training should emphasise the importance of self-awareness as non-patient variables can potentially impact assessments.^29^ There is much we can learn from police education. For example, included articles described the importance of patient-centred training, operational feasibility, and the need for experiential learning. They also acknowledged the important role that senior colleagues, patients, and carers play in training.^25,30–32^

### Legislation

All of the countries included have specific mental health detention legislations, with variations in law between and even within countries in the UK. Findings identified legislative knowledge gaps across disciplines.^22,33–36^ Scottish surveys highlighted gaps in essential legislative knowledge among GPs.^10,36^ There was a call for training to incorporate foundational legal knowledge, recognising that training needs to create opportunities for knowledge application and discussing complex cases. This is important given a survey among doctors in the US demonstrated how inexperience can potentially be linked to inappropriate involuntary commitment.^37^

Findings suggested the need for mandatory and refresher training courses to maintain necessary skills; however, none of the articles gave clear recommendations on the frequency and approach for refresher training. Suggestions for training development included the use of clinical vignettes, small group sessions, discussion of real-life scenarios, multidisciplinary teaching, and the opportunity to learn from seniors with experience.^34,37,38^ A survey among GPs suggested practical training in legislation application. They found previous practical experience was associated with better current knowledge (*P* = 0.0074) and confidence in using relevant legislation (*P* = 0.0005).^10^ It was also suggested that decision-making aids could be helpful for more complex cases.^39,40^ It was recognised that such tools need to be user-friendly, with tailored training before implementation.^40^

### Unmet training needs and wider difficulties with the process

Lack of a timely, structured, coordinated approach to assessment and admission tends to be associated with negative outcomes that are drivers for change. For example, many professionals felt at risk during these assessments as the process can be chaotic and stressful.^9,41,42^ A survey among GPs found that one-third (*n* = 16) expressed concerns about their own personal safety and admitted this fear would deter them from detaining a patient.^42^ Ambulance delays created additional safety concerns with an increased risk of a patient absconding.^9,42^ GPs acknowledged the wider complexities and expressed concerns about the legal focus on the process rather than the patient needing help.^9^ Articles highlighted challenges of multidisciplinary working and potential benefits of interdisciplinary training.^2,35,43,44^ Such training could increase understanding of respective roles and ensure a more cohesive response.

Potential areas for training development included training on self-defence, documentation, communication skills, and opportunity for discussion of complex cases, particularly those that did not meet detention criteria.^2,25,28,45^ Articles acknowledged the range of situational and emotional factors encountered that can only really be learnt through practical experience and listening to seniors, service users, and carers with experience of this emergency.^6–8^ Developments in training need to be complemented by wider service improvements and alternatives to hospital admission.^46^ Suggested areas for improvement included the need for more straightforward and standardised paperwork, more efficient transportation to hospital, post-assessment debriefing, as well as improvements in supportive resources and services available for patients and their families.^7,28,41,47,48^

### Consultation with professionals

The findings of the scoping review resonated strongly with professionals included in the consultations. It was suggested that current training is limited. All professionals were keen to engage in training and called for experiential learning, which provides opportunity for practical experience and discussion of complex cases. This phase of the review was an extensive qualitative study in its own right; therefore, a separate thematic analysis of this work is planned to be conducted, which will be published at a later stage.

## Discussion

### Summary

This review confirms a training gap for GPs and professional colleagues involved in mental health detention assessments.^2,10^ To date, training needs have been overshadowed by discussions about service issues and process difficulties. Professionals involved acknowledge this is a complex crisis and are keen to engage in suitable training.^2,10,28,49^ This review maps and consolidates key messages from the literature to inform training development moving forward.

### Strengths and limitations

An important strength of the work is the iterative development of the scoping review focus. Given the apparent lack of literature focused specifically on education and training in this area, particularly in the primary care setting, the scoping net was cast wide, capturing a range of insightful articles across different settings and professional groups. Including discussion articles written by professionals with experience meant key messages and challenges of the process could be captured that an exclusive focus on empirical research would have missed. The interdisciplinary consultation exercise supports the review findings and adds depth of understanding and clinical relevance to the review.

A scoping review is, by definition, descriptive, and does not critically appraise included literature. This inevitably means that isolated issues and opinions are considered alongside findings from more methodologically robust studies. It is acknowledged that no search strategy is perfect and it is plausible that some relevant articles may not have been captured owing to vagaries of titles, abstracts, language, or search syntax. Despite best efforts, some full texts were inaccessible, thus the study has potentially missed capturing some landscape breadth. Every review research team makes decisions around inclusion. A different team, in a different place, may have made different decisions and come up with a different map. This is the authors' landscape map and is offered with that implicit limitation for others to use as reference point for future research in this area.

### Comparison with existing literature

No other review articles were identified that focused specifically on training development in this area. This review maps and consolidates available literature and, in doing so, highlights key factors that should be considered in the development of meaningful, educational approaches. Literature to date focuses on describing the challenges of the process, gaps, and potential benefits of training.^6^ Many discussion articles were identified highlighting complexity of the process, suggesting a range of factors for consideration in the development of training in this area.^23,34,46^ This review takes things further by synthesising available evidence, rendering it more accessible to practitioners. Articles were encountered highlighting potential benefits of interdisciplinary teaching and discussion.^2,16^ The present review supports this approach; there is much that can be learnt from each other. The importance of learning from those with experience and ensuring patient-centred care are other key messages.^1,2,7^

Research articles describing and reviewing tried-and-tested approaches in this area are limited. Coverage of theory alone is unlikely to adequately prepare professionals for this emergency.^6^ There is a call for practical training that enables discussion of complex cases and interdisciplinary teaching.^2,37^

### Implications for research and practice

This review shows that education and research in this area lags behind clinical need. There is a need for the development, implementation, and review of different training approaches, especially in primary care settings. Development and implementation of any educational approach needs careful planning and consideration. The review provides educators with a strong foundation on which to develop meaningful educational approaches. Training approaches should be flexible to accommodate local variability in legislation and services; however, there are many common principles in the training process that should be standardised. For example, the findings suggest that training needs to bridge the theory–practice gap. GPs and respective colleagues are keen for practical, interdisciplinary, patient-centred training, which provides a safe space for discussing complex cases. There is also a recognised need to learn from those with experience of the process, for example, senior colleagues, carers, and patients. Moving forward, these pearls of wisdom need to be integrated into training approaches.
